# Knowledge and Behavioral Effects in Cardiovascular Health: Community Health Worker Health Disparities Initiative, 2007–2010

**DOI:** 10.5888/pcd11.130250

**Published:** 2014-02-13

**Authors:** Margarita Hurtado, Jovonni R. Spinner, Manshu Yang, Christian Evensen, Amy Windham, Gloria Ortiz, Rachel Tracy, Edward Donnell Ivy

**Affiliations:** Author Affiliations: Margarita Hurtado, Manshu Yang, Christian Evensen, Amy Windham, American Institutes for Research, Washington, DC; Gloria Ortiz, Rachael Tracy, Edward Donnell Ivy, National Institutes of Health, Bethesda, Maryland.

## Abstract

**Introduction:**

Cardiovascular disease is the leading cause of death in the United States, and disparities in cardiovascular health exist among African Americans, American Indians, Hispanics, and Filipinos. The Community Health Worker Health Disparities Initiative of the National Heart, Lung, and Blood Institute (NHLBI) includes culturally tailored curricula taught by community health workers (CHWs) to improve knowledge and heart-healthy behaviors in these racial/ethnic groups.

**Methods:**

We used data from 1,004 community participants in a 10-session curriculum taught by CHWs at 15 sites to evaluate the NHLBI’s health disparities initiative by using a 1-group pretest–posttest design. The curriculum addressed identification and management of cardiovascular disease risk factors. We used linear mixed effects and generalized linear mixed effects models to examine results.

**Results:**

Average participant age was 48; 75% were female, 50% were Hispanic, 35% were African American, 8% were Filipino, and 7% were American Indian. Twenty-three percent reported a history of diabetes, and 37% reported a family history of heart disease. Correct pretest to posttest knowledge scores increased from 48% to 74% for heart healthy knowledge. The percentage of participants at the action or maintenance stage of behavior change increased from 41% to 85%.

**Conclusion:**

Using the CHW model to implement community education with culturally tailored curricula may improve heart health knowledge and behaviors among minorities. Further studies should examine the influence of such programs on clinical risk factors for cardiovascular disease.

## Introduction

Cardiovascular disease (CVD) is the leading cause of death in the United States, accounting for 32% of deaths (1). Disparities in the prevalence of CVD and associated risk factors (eg, hypertension, high blood cholesterol, smoking, overweight, and obesity) have been documented in racial and ethnic minority populations (2–4). African Americans have the highest rate of hypertension in the United States (44% vs 29% among non-Hispanic whites) (2), and Hispanics and African Americans have the highest rates of obesity (3). American Indians have the highest prevalence of coronary heart disease, which affects 11.6% of their population (4), and Filipinos are significantly more likely than non-Hispanic whites to have hypertension (5).

To address these health disparities, the National Heart, Lung, and Blood Institute (NHLBI) created the Community Health Worker (CHW) Health Disparities Initiative, drawing on science-based materials, resources, and educational programs to improve health in minority and underserved communities. A key strategy uses a CHW-driven, community-based, participatory approach to deliver community education sessions to promote heart health and reduce health disparities. CHWs are known by many names, such as *promotores de salud*, community health representatives, or community educators; however, we refer to them here as CHWs.

**Figure 1:**
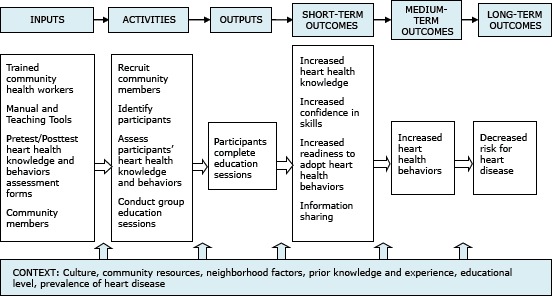
Logic model for the community health worker-led heart health program describing the inputs, activities, outputs, and outcomes of the National Heart, Lung, and Blood Institute’s Community Health Worker Health Disparities Initiative.

Before beginning the community education sessions, CHWs are trained on NHLBI’s heart health curricula. The curricula have a common core, and each curriculum component is designed to be culturally and linguistically appropriate for African Americans, American Indians, Filipinos, or Hispanics. The curricula are designed to increase knowledge and improve lifestyle behaviors associated with cardiovascular health through culturally competent (6) community education activities. Supporting materials include picture cards, recipe books, and risk factor booklets that are used to reinforce key messages (7). Figure 1 shows the logic model for the community education strategy.

Smaller CHW programs in Hispanic communities in Arizona, Texas, and California increased knowledge (8) and improved heart health behaviors (9,10). Programs using NHLBI’s curriculum in conjunction with clinical management have also demonstrated reductions in heart disease risk factors, including improvements in low-density lipoprotein cholesterol level, triglyceride level, blood pressure, weight, and glycated hemoglobin level (11). However, other studies and systematic reviews of CHW programs have shown mixed evidence of CHWs effectiveness in the prevention and control of CVD (12,13).

Our study assesses effects on heart health knowledge, CVD risk factor behaviors, confidence in preparing heart healthy food, and readiness to change behavior among 1,004 community participants in group education sessions. Our study was conducted in various settings, including community-based organizations, clinics, training centers, tribal organizations, and public housing.

## Methods

### Setting and intervention

From 2007 through 2010 trained CHWs delivered NHLBI’s heart health curricula to small groups at 15 sites across the United States. The sites were selected through a competitive process and included community-based organizations, clinics, and tribal organizations in African American, American Indian, Filipino, and Hispanic communities. A curriculum was developed tailored to each of these races/ethnicities. Participating sites recruited adults aged 18 years or older from the general population in their communities. Participants attended 10 sessions that covered identifying risk factors for heart disease, recognizing the signs of heart attack, preparing healthy meals, eating healthy on a budget, controlling blood pressure, controlling weight, managing diabetes, lowering high blood cholesterol, increasing physical activity, and decreasing smoking. The sessions were taught weekly or biweekly and lasted approximately 2 hours. CHWs collected information from participants before and after completing all the sessions by using self-report instruments to assess changes in heart health knowledge, attitudes, and behaviors.

### Evaluation measures

We used an observational 1-group, pretest–posttest design to examine changes in outcomes between baseline and completion of the program. The data collection instrument, called “My Health Habits,” was a survey included as part of each curriculum. Data were routinely collected as part of the community education program to allow sites to monitor their own outcomes and were later used for our evaluation. The National Institutes of Health Office of Management and Budget gave the survey instrument a clinical exemption for collection of participant data. NHLBI contracted with the American Institutes for Research to analyze the data for this summary outcome evaluation. The “My Health Habits” survey was used to assess 5 outcome domains:

**1. Heart health knowledge.** The 13 knowledge items were summed into an average percentage score for each participant where the score indicated the percentage of items answered correctly.

**2. CVD food-related risk factor behaviors.** The 25 food-related behaviors frequency items were scored on a 4-point scale (1 = never, 2 = sometimes, 3 = most of the time, and 4 = always). An overall summed average frequency of the 25 items was calculated, as well as 3 subscales addressing salt and sodium consumption (9 items), cholesterol and fat consumption (8 items); and weight management behaviors (8 items).

**3. Physical activity.** Nine items assessing frequency and intensity of physical activity were used to categorize participants as physically active or not physically active. “Physically active” was defined as engaging in moderate to vigorous aerobic physical activity during leisure time at least 2 days a week for more than 30 minutes each day. Otherwise, participants were classified as not physically active.

**4. Confidence in preparing heart-healthy food.** This was a 1-item measure scored on a 4-point scale (1 = not confident, 2 = somewhat confident, 3 = confident, and 4 = very confident). The item was dichotomized by combining the 2 lower categories (not confident and somewhat confident) and 2 higher categories (confident and very confident) on the basis of its bimodal distribution.

**5. Stages of change.** The stage of change measure is based on the transtheoretical model, which assesses an individual’s readiness for change along the following 5-stage continuum: precontemplation, contemplation, preparation, action, and maintenance (14). This survey measure is presented to respondents as a scenario that describes family members at each stage along the continuum and asks them to indicate who they identify with most. The grandmother, for example, represented the action stage and was described as someone who “takes classes to learn how to improve her health and puts into practice what she learns. . . . She uses recipes to cook healthfully and takes walks every day.” We dichotomized this 5-point measure by combining the first 3 stages of behavior change (precontemplation, contemplation, and preparation) and the last 2 stages (action and maintenance). This measure was not included in the survey administered at Filipino sites.

### Data analysis

For each site, data from the pretests and posttests were linked by a participant identification number when one was available. We used linear mixed effects models (15) for continuous outcomes and generalized linear mixed models (16) for categorical outcomes to estimate changes in outcome measures at the start and end of program implementation. These models account for potential correlations among participants within the same study site or from repeated measures for the same participant. For respondents with missing scores at the pretest but not the posttest, or vice versa, the observed portion of their data was kept in the analysis. By using the Maximum Likelihood estimation method in the analytic models, we obtained regression coefficients that were adjusted for the missing values by assuming that the data were missing at random. The parameter estimates from the Maximum Likelihood estimation are very similar to those obtained from the multiple imputations. The former, however, is more efficient because adjusted regression coefficients and standard errors can be obtained without generating complete data sets with imputed pretest or posttest scores (17). Demographic and CVD risk characteristics (eg, age, sex, told by a health professional they had diabetes, family history of heart disease) and the setting of program implementation (ie, clinic, community based organization [CBO], or other type of site) were included in the models as control or predictor variables. When possible, results for each curricula tailored to a racial/ethnic group were reported. Associations between participant characteristics related to CVD (eg, having diabetes or a family history of heart disease) and outcomes were examined by using multiple regression models. Statistical analyses were conducted using SAS version 9.2 (SAS Institute, Inc, Cary, North Carolina).

## Results

### Participant and site characteristics

The program was implemented most often through CBOs, which served 44% of participants, followed by clinics, which served 19% (Table 1). The remaining participants attended sessions in other community settings such as schools or apartment buildings. Seventy-five percent of participants were women, and their average age was 48 years. Approximately 50% of participants were Hispanic, 35% African American, 7% American Indian, and 8% Filipino. In terms of CVD risk factors, 23% had been told by a health care professional that they had diabetes and 37% reported a family history of heart disease.

**Table 1:** Participant Characteristics of the National Heart, Lung, and Blood Institute’s Community Health Worker Disparities Initiative, by Curriculum, 2007–2010

Characteristic of Curriculum Site	Hispanic	African American	American Indian	Filipino	Total
**Number of participants, n (%)**	501 (50)	354 (35)	67 (7)	82 (8)	1,004
**Number of sites**	7	3	2	3	15
**Type of organization, n (%)**					
Community-based organization	117 (23)	211 (60)	43 (64)	70 (85)	441 (44)
Clinic	148 (30)	19 (5)	24 (36)	4 (5)	195 (19)
Other	236 (47)	124 (35)	0	8 (10)	368 (37)
**Sex, n (%)**					
Female	429 (86)	213 (60)	55 (82)	53 (65)	750 (75)
Male	72 (14)	141 (40)	12 (18)	29 (35)	254 (25)
**Average age, y**	41	58	42	63	48
**Reported being told by a health care professional that they had diabetes, n (%)**	108 (22)	98 (28)	17 (25)	10 (12)	233 (23)
**Reported a family history of heart disease, n (%)**	221 (44)	126 (36)	24 (36)	4 (5)	375 (37)

### Response rates

Overall, 1,004 total participants attended the education sessions. Of these, 849 participants (85%) completed both the pretest and posttest surveys later used for the evaluation; 123 (12%) completed only the pretest, and 32 (3%) completed only the posttest. The percentage of participants who had matched data from the pretest and posttest varied across curricula: 99% for the African American curriculum, 92% for the Hispanic curriculum, 40% for the American Indian curriculum, and 13% for the Filipino curriculum. The percentage of Filipino participants that had both a pretest and posttest that could be linked was low because 2 of the 3 Filipino sites did not use the unique identification numbers needed for linkage. In our analysis we kept the observed portion of data for those respondents whose data could not be linked between pretest and posttest and those who had missing scores at the pretest but not the posttest, or vice versa.

### Overall outcomes

We adjusted the outcomes at pretest and posttest for demographic and CVD risk characteristics (ie, age, sex, personal history of diabetes, and family history of heart disease), and whether the program was implemented through a clinic, CBO, or other type of site (Table 1). We calculated overall results representing summary estimates of changes in the outcomes from pretest to posttest and curriculum-specific results.

**Heart health knowledge.** The percentage of correct answers on the heart health knowledge questions significantly increased, on average, from 48% to 74% pretest to posttest (*P* < .001) (Table 2).

**Table 2:** Knowledge and Behavioral Effects of the National Heart, Lung, and Blood Institute’s Community Health Worker Health Disparities Initiative, “My Health Habits” Survey Outcomes, 2007–2010

Measure	Curriculum	N	Pretest Mean (SE)^a^	Posttest Mean (SE)^a^	*P* value^b^
Heart health knowledge^c^	All curricula combined	1,004	48% (3%)	74% (3%)	<.001
	African American	354	44% (3%)	77% (3%)	<.001
	American Indian	67	63% (4%)	73% (4%)	.002
	Filipino	82	39% (10%)	66% (9%)	.02
	Hispanic	501	49% (2%)	71% (2%)	<.001

Frequency of engaging in healthy food-related behaviors^d^	All curricula combined	1,004	2.5 (0.1)	2.9 (0.1)	<.001
	African American	354	2.6 (0.1)	2.9 (0.1)	<.001
	American Indian	67	2.4 (0.1)	2.6 (0.1)	.02
	Filipino	82	2.7 (0.2)	2.9 (0.2)	.18
	Latino	501	2.4 (0.1)	2.8(0.1)	<.001

Physically active^e^	All curricula combined	1,004	33% (8%)	65% (8%)	<.001
	African American	354	68% (6%)	91% (3%)	<.001
	American Indian	67	8% (5%)	35% (17%)	.006
	Filipino	82	58% (33%)	58% (42%)	.98
	Latino	501	22% (4%)	47% (6%)	<.001

Confidence in preparing heart-healthy food^f^	All curricula combined	1,004	40% (8%)	88% (4%)	<.001
	African American	354	51% (5%)	97% (1%)	<.001
	American Indian	67	27% (9%)	52% (15%)	.11
	Filipino	82	33% (25%)	100% (2%)	.57
	Latino	501	51% (5%)	87% (3%)	<.001

Stage of change^g^	All curricula combined (excluding Filipino^h^)	922	41% (6%)	85% (4%)	<.001
	African American	354	61% (6%)	94% (2%)	< .001
	American Indian	67	51% (17%)	42% (16%)	.64
	Latino	501	38% (6%)	85% (4%)	< .001

Abbreviation: SE, standard error.

a Values indicate mean outcome scores, followed by the standard errors in parentheses, adjusted for participant age, sex, and personal history of diabetes; family history of heart disease; the type of curriculum used; and the type of location where the community education took place. The SEs of outcomes are reported in percentage points for the Knowledge outcome (based on a scale of 0% to 100%) and reported on a scale of 1 to 4 for the Food-Related Behaviors outcomes. The Physical Activity, Confidence, and Stages of Change scores are binary outcomes. The SEs are reported using a percentage point scale.

b *P* values were calculated by using linear mixed effects models for heart health knowledge and diet-related behaviors and by using generalized linear mixed models for physical activity, confidence in healthful cooking, and stage of behavior change.

c Knowledge scores represent the percentage of correct responses.

d Food-related behavior frequency scores represent the average score on a scale from 1 to 4, with 1 = never, 2 = sometimes, 3 = most of the time, and 4 = always.

e Percentages of participants who reported they were physically active. “Physically active” was defined as engaging in moderate to vigorous aerobic physical activity during leisure time at least 2 days a week and for more than 30 minutes per day.

f Percentage of participants who reported they were confident or very confident.

g Stage of change assesses an individual’s readiness for change along the following 5-stage continuum: precontemplation, contemplation, preparation, action, and maintenance. Values represent the combined percentage of participants in the action and maintenance stages of change.

h Filipino sites did not report on the stage of change measure.

**Figure 2:**
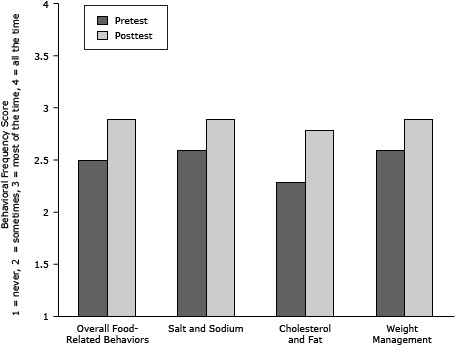
Frequency scores of self-reported food-related behaviors associated with cardiovascular health, overall and in each of the 3 specific areas before (pretest) and after (posttest) attending educational sessions, National Heart, Lung, and Blood Institute’s Community Health Worker Health Disparities Initiative. Bars indicate behavioral frequencies on a 1 to 4 scale (1 = never, 2 = sometimes, 3 = most of the time, 4 = always). Pretest–posttest changes are significant at *P* < .001. **Table:** 

**CVD food-related risk factor behaviors.** The frequency of self-reported food-related behaviors associated with cardiovascular health increased significantly on the overall score, from 2.5 to 2.9 on a 1 to 4 scale, and in each of the 3 subdomains: salt and sodium consumption, cholesterol and fat consumption, and weight management (*P* < .001) (Table 1)(Figure 2).

Measure	Pretest Mean	Posttest Mean
**Overall food-related behaviors**	**2.5**	**2.9**
**Salt and sodium**	**2.6**	**2.9**
**Cholesterol and fat**	**2.3**	**2.8**
**Weight management**	**2.6**	**2.9**

**Physical activity.** The percentage of participants classified as physically active increased from 33% to 65% pretest to posttest (*P* < .001) (Table 2).

**Confidence.** A significantly greater proportion of participants reported being confident or very confident in being able to prepare heart healthy foods for themselves and their families posttest (88%) than on pretest (40%) (*P* < .001) (Table 2).

**Stages of change.** Eighty-five percent of participants, on the basis of the scenarios presented, self-identified as being in the action or maintenance stage of change at posttest compared with 41% at pretest (*P* < .001) (Table 2).

**Significant predictors of changes in knowledge and behavior.** Age and personal history of diabetes were found to be significant predictors for the magnitude of change in the overall score of heart health behaviors. Younger participants and those without a known history of diabetes made the greatest gains in the adoption of heart-healthy behaviors. Personal history of diabetes and family history of heart disease were significant predictors of heart health knowledge. Participants who did not have diabetes or did not know their status and those who did not say they had a family history of heart disease had larger improvements in heart health knowledge than those who responded affirmatively to either of these questions.

### Participant’s program satisfaction

More than 97% of participants were satisfied or very satisfied with the curriculum. Most indicated they shared the information learned with their friends (81%), family (95%), coworkers (24%), or with other associates (21%).

### Curriculum-specific results

We analyzedthe results to determine whether they varied across groups using the different curricula. The American Indian and Filipino study sites had considerably fewer participants (67 and 82, respectively) than African American or Hispanic sites (354 and 501, respectively), so these results should be interpreted with caution.

**Knowledge.** Heart health knowledge increased significantly with all 4 curricula, ranging from 22 percentage points for the African American curriculum to 33 percentage points for the Hispanic curriculum.

**Behavior.** Participants in all 4 curricula had improvements in frequency of self-report of adopting healthy food-related behaviors that ranged from 0.2 points for the American Indian curriculum to 0.4 points for the Hispanic curriculum. This increase was significant for all curricula except Filipino sites, where the sample size was small and the individual pretest and posttest measures could not be linked. Although the unlinked cases were included in the analyses, the comparisons in the unmatched analyses are less precise and have less statistical power to detect differences than comparisons with matched cases. Self-reported physical activity also increased significantly in all groups except those using the Filipino curriculum.

**Confidence.** Participants in the African American and Hispanic curricula reported significantly higher levels of confidence in their ability to prepare heart healthy foods after participating in the program. Participants in the Filipino and American Indian curricula also reported higher confidence, but levels were not significant.

**Stage of change.** African American and Hispanic participants had a significantly larger proportion of participants at the action or maintenance stage of change at posttest than at pretest compared with American Indians, Alaska Natives and Filipinos. Results were not significant for American Indian participants. The Filipino sites did not report on the stage-of-change measure.

## Discussion

This study is the largest evaluation of a CHW-based program to date. It assesses the effect across 4 different minority groups, unlike other interventions that target specific ethnic groups or are not culturally grounded. Findings from this study provide evidence that CHW-led interventions have a positive effect on heart health knowledge and self-reported health behaviors in minority and underserved communities. The CHW model has been used historically to bridge the gap between the health care system and its clients. CHWs have been effective at providing community education and at helping patients navigate the system, thus promoting improved health outcomes (18). The results observed in this evaluation are in line with other studies that used a similar research design (11,19). With few exceptions, positive results were observed across all curricula. Community participants generally increased their heart health knowledge and improved in self-reported health behaviors (eg, increasing physical activity) that can reduce CVD risk. Additionally, participants progressed to a stage of change more closely aligned with practicing heart healthy behaviors on a regular basis. Given the successes of the experiences documented here, consideration should be given to expanding this program in similar minority and underserved communities. More rigorous evaluations are needed to further examine CHW’s abilities to effectively improve behavior change as well as improve clinical outcomes (eg, blood pressure). In addition, the curricula may need to be updated to reflect the current science (eg, revised physical activity and nutrition guidelines) and implemented with different methods (eg, online education) to keep up with rapid changes in technology.

Because it was based on a convenience sample, our evaluation did not include a control group; therefore, the observed changes cannot be attributed solely to the intervention and are not generalizable beyond the study population. Anecdotally, program implementation strategies varied across sites (eg, duration and number of sessions, teaching style of CHWs) to accommodate resource limitations and community needs. These and other issues could have contributed to the variability in results across the 4 curricula. Lack of unique identification numbers from the Filipino sites limited the ability to match pretest and posttest scores for individuals. Although the analysis included these individuals by applying appropriate methods to deal with missing data, results for these sites might have been more robust had the data been matched. Finally, the data collected were self-reported and subject to recall and social desirability biases.

Despite limitations, the changes we observed were large, significant, and consistent across settings and racial/ethnic-specific curricula applied to over 1,000 participants. The results add to the evidence that the CHW model is effective in improving heart health knowledge and behaviors and can move participants along a continuum of being able to implement and sustain positive heart health behaviors. Robust predefined evaluation studies using a control group and measuring clinical outcomes would contribute more evidence on the effectiveness of the CHW model to reduce risk factors for CVD. It also would be valuable to examine the content and methods of the CHW training program and to link training quality and program fidelity to outcomes for community participants.

Cardiovascular health disparities persist among racial and ethnic minority groups. Programs are needed that are culturally and linguistically tailored to these groups and are easy to implement with limited resources. The curricula used in this intervention program met these criteria and were well received by the CHWs, community participants, and organizations. The curricula can be more widely applied to help reduce racial and ethnic disparities in cardiovascular health. NHLBI continues to expand implementation of the curricula through building partnerships with various organizations (eg, public housing authorities, academia, CBOs, clinics) in an effort to help reduce CVD disparities. For example, another study of 97 Filipino participants showed promising results for an expanded version of the program where individuals screened for being at risk for heart disease were followed and managed in a clinic setting in addition to participating in group education activities led by CHWs (19). Findings from clinical trials of similar interventions provide additional evidence of the effectiveness of this type of program in preventing CVD and improving health outcomes (20). Further development and testing of similar efforts using CHWs to decrease risk factors for CVD and improve outcomes are needed to support widespread implementation of this type of approach (21). This study adds to the body of literature by showing that CHWs can be part of the solution to address heart health disparities by using NHLBI's curricula in minority communities.
